# *IPA1* improves drought tolerance by activating *SNAC1* in rice

**DOI:** 10.1186/s12870-023-04062-9

**Published:** 2023-01-25

**Authors:** Feihe Chen, Haomin Zhang, Hong Li, Ling Lian, Yidong Wei, Yuelong Lin, Lanning Wang, Wei He, Qiuhua Cai, Hongguang Xie, Hua Zhang, Jianfu Zhang

**Affiliations:** 1 Rice Research Institute, Fujian Academy of Agricultural Sciences, Fuzhou, 350018 China; 2 College of Agriculture, Fujian Agriculture and Forestry University, Fuzhou, 350002 China; 3 State Key Laboratory of Ecological Pest Control for Fujian and Taiwan Crops/Key Laboratory of Germplasm Innovation and Molecular Breeding of Hybrid Rice for South China, Ministry of Agriculture and Affairs, P.R. China/Incubator of National Key Laboratory of Germplasm Innovation and Molecular Breeding between Fujian and Ministry of Sciences and Technology/Fuzhou Branch, National Rice Improvement Center of China/Fujian Engineering Laboratory of Crop Molecular Breeding/Fujian Key Laboratory of Rice Molecular Breeding, Fuzhou, 350003 China

**Keywords:** IPA1, Rice (*Oryza sativa* L.), Drought stress, *SNAC1*, Reactive oxygen species (ROS)

## Abstract

Drought is a major abiotic stress to rice (*Oryza sativa*) during growth. Ideal Plant Architecture (*IPA1*), the first cloned gene controlling the ideal plant type in rice, has been reported to function in both ideal rice plant architecture and biotic resistance. Here, we report that the *IPA1/OsSPL14*, encoding a transcriptional factor, positively regulates drought tolerance in rice. The *IPA1* is constitutively expressed and regulated by H_2_O_2_, abscisic acid, NaCl and polyethylene glycol 6000 treatments in rice. Furthermore, the I*PA1*-knockout plants showed much greater accumulation of H_2_O_2_ as measured by 3,3′-diaminobenzidine staining in leaves compared with WT plants. Yeast one-hybrid, dual-luciferase and electrophoretic mobility shift assays indicated that the IPA1 directly activates the promoter of *SNAC1*. Expression of *SNAC1* is significantly down-regulated in *IPA1* knockout plants. Further investigation indicated that the *IPA1* plays a positive role in drought-stress tolerance by inducing reactive oxygen species scavenging in rice. Together, these findings indicated that the *IPA1* played important roles in drought tolerance by regulating *SNAC1*, thus activating the antioxidant system in rice.

**Supplementary Information:**

The online version contains supplementary material available at 10.1186/s12870-023-04062-9.

## Introduction

Drought is one of the most important factors affecting crop production and has severe effects on food security [1]. Breeding varieties to improve their agronomic traits, such as ideal plant structure, is a key factor in increasing food production [2]. Several key genes controlling agronomic traits have been cloned, including those encoding several transcription factors of the SQUAMOSA-PROMOTER BINDING PROTEIN-LIKE (SPL) gene family [3]. The SPL gene family regulates the structure of rice plants [4] and controls flowering time [5]. In *Arabidopsis*, 11 of the 17 SPL genes are targets of the microRNA (miRNA) miR156 [5], and in rice 11 of 19 SPL genes are targets of miR156 [3, 6].

Previous research of miR156 shows that it is an important regulatory element in a variety of plant biological processes, including plant growth, development, environmental stress response and defense [7]. MiR156 and its target gene SBP/SPL have major roles in plant development [8, 9]. Plants coordinate plant development and abiotic stress tolerance through the miR156-SPL9 pathway [10]. MiRNA expression decreases, and SPL expression increases throughout development [11]. Previous studies have shown that MIR156 expression is induced, and SPL gene expression is suppressed, when plants are exposed to drought stress, and that MIR156 expression decreases, and SPL gene expression begins to increase, when normal growth conditions are restored; this MIR156-SPL mediated abiotic stress response is functionally conserved in rice [12].

The *SPL* genes have been studied primarily for their functions in growth and development. In rice, overexpression of OsSPL16 promotes cell division and seed filling [13]. Overexpression of OsSPL17 decreases tillering in rice plants [14]. OsSPL14 is an IDEAL PLANT ARCHITECTURE1 (IPA1) gene controlling tillering in rice. The concept of ideal plant architecture was first identified through a mutation in a recognition site on miRNA that alters the transcription of *IPA1* gene, thus decreasing tiller number, and resulting in taller plants and more panicle branches [15]. Full gene expression profiling has indicated that IPA1 directly binds GTAC, the core motif of the SBP-box, and influences plant growth and development by regulating OsTB1 and DEP1 [16]. Since then, *IPA1* has been identified to play a role in abiotic stresses in rice [17, 18]. Phosphorylated IPA1 binds the promoter of WKRY45 and enhances resistance to disease [17]. Overexpression of *IPA1* increases disease resistance to bacterial blight in rice [18]. Recent studies have shown that *IPA1* increases drought tolerance in rice seedlings through the abscisic acid (ABA) pathway [19]. In conclusion, *IPA1* plays a role not only in regulating plant structure but also in plant resistance to abiotic stresses and diseases.

Under stressful conditions, the balance between intracellular ROS production and clearance is disrupted [20, 21]. Thus, exposure of plants to various environmental stresses can lead to excessive production of ROS [22]. Drought is a limiting factor for rice production and can lead to overproduction of reactive oxygen species. However, when ROS accumulates in excess, it triggers progressive oxidative damage, leading to retarded rice growth and eventually cell death [23]. Several studies have shown that increasing the expression of ROS scavenging-related genes can increase tolerance to drought stress. For example, overexpression of *SNAC3* regulates ROS homeostasis by regulating the expression of ROS scavenging genes, thereby increasing drought tolerance in rice [24]. Overexpression of *OSLG3* improves drought tolerance in rice by enhancing ROS scavenging enzyme activity and significantly reducing ROS accumulation [25]. Therefore, an effective way to improve tolerance to drought-induced oxidative damage is to increase the efficiency of antioxidant activity in rice [26]. Therefore, the relationship between drought stress and ROS homeostasis is critical. In the present study, we generated *IPA1* knockout transgenic rice and analyzed its function related to drought stress in rice. Research on *IPA1* currently focuses on rice plant structure and biotic stresses, whereas studies of abiotic stresses are relatively scarce in rice. Here, *IPA1* is demonstrated to positively regulate drought tolerance by affecting reactive oxygen species (ROS) content in rice. Previously, we found that *IPA1* regulates many drought-related transcription factors [16, 19], and through screening and validation we determined that *IPA1* directly regulates *SNAC1* and might regulate drought resistance in rice，and suggest a new role for *IPA1* in the involvement of drought stress in rice.

## Materials and methods

### Plant material and growth conditions

The rice varieties FH7185 and MH86 (*Oryza sativa* L*.*) from Rice Research Institute, Fujian Academy of Agricultural Sciences, China was used in this study. The germinated seeds were planted in soil, and the seedlings were grown under standard greenhouse conditions (16-h light at 28 °C/8-h dark at 26 °C).

### Vector construction and rice transformation

The full-length cDNA of *IPA1* was amplified from rice cultivar FH7185 by RT-PCR, and the sequence-confirmed PCR fragment was inserted into the pHUE411 vector under the control of. The vectors were constructed by insertion of synthesized oligonucleotides into the *BsaI* site of the vector pYLCRISPR/Cas9, which contains a codon-optimized Cas9 driven by a maize ubiquitin promoter for knockout, which was introduced into the *A. tumefaciens* strain EHA105. Agrobacterium-mediated transformation of rice (*Oryza sativa* L. subsp. indica FH7185) was performed according to (References) [27]. The plasmid pCAMBIA1300-OsSPL14 was introduced into mature rice (*O. sativa* L. indica cultivar‘MH86’) embryos with Agrobacterium-mediated transformation. Stably inherited transgenic plants possessing single copy insertions of the transgene were selected and used in this study.

### Expression pattern of *IPA1* under abiotic stress

Selecting seedlings at the three-leaf stage for treatment. Polyethylene glycol (PEG) 6000 concentration of 20%, ABA and GA at a concentration of 100 μM, NaCl at a concentration of 100 mM. PEG treatment times of 0 h, 1 h, 2 h, 4 h, 8 h, 12 h, 24 h and 36 h. ABA, GA and NaCl treatment times of 0 h, 1 h, 3 h, 6 h, 12 h, 24 h and 36 h. Sampling by time point, each group with clear water as blank control, three biological replicates per group, three plants per replicate.

### Drought treatment of plants at the seedling stage for rice

T4 generation rice plants were used in the experiment. The drought stress experiment was conducted in plastic buckets with soil. For assays of drought treatment, 12 plants of each line were used in each replicate, with three replicates for each line. The plants grew in buckets until the five-leaf stage, after which irrigation stopped for 14 days. After recovery with water for 7 days, the survival rate was measured. Drought stress was simulated in a plastic bucket with 0.5 m soil depth.

In dehydration treatment, In the dehydration treatment, WT (MH86) and IPA1-OE plants of uniform growth were transplanted onto 96-well PCR plates and hydroponically grown with nutrient solution. The plants grew until the five-leaf stage, after which polyethylene glycol (PEG) 6000 concentration of 25% treatment for 10 days.

### RNA extraction, quantification and RT-qPCR analysis

Rice leaf RNA was extracted from transgenic rice plants and wild-type FH7185 rice plants. Total RNA was extracted with a TriZol Up kit (TransGen Biotech, China) according to the manufacturer’s protocol. An RNA reverse-transcription kit with gDNA Remover (Toyobo, Japan) was used to generate cDNA for 10 min at 25 °C, 120 min at 37 °C and 5 min at 85 °C. qRT-PCR was performed with ChamQ Universal SYBR qPCR Master Mix (Vazyme, China) on an ABI Prism 7500 real-time PCR system.

### Yeast one-hybrid assays

The coding region of *IPA1* was amplified and cloned into the PJG4–5 prey vector. The promoters of *SNAC1* were amplified and cloned into pLaczi-2u bait vectors. The prey vectors were co-transformed with bait vector into EYG48 strain. The transformed yeast cells were grown on SD/−Trp/−Ura medium and then applied to yeast plates containing 5-bromo-4-chloro-3-indolyl β-D-galactoside. Interactions were screened on the basis of presence of blue pigment. The empty vector pJG4–5 and recombinant pLacZi vectors were co-transformed as negative controls.

### Electrophoretic mobility shift assay (EMSA)

The full length *IPA1* coding sequence was fused into the pMAL-C5X vector, and the fusion protein was expressed in *Escherichia coli* and purified. The double-stranded Cy5.5-labeled probes used in this assay were synthesized by Biosun (China). The EMSA was performed with an EMSA/Gel-Shift Kit (Beyotime, China) according to the manufacturer’s instructions. Briefly, 2 mg of purified MBP or MBP-IPA1 protein was added to the binding reaction and incubated for 20 min at 25 °C in a thermal cycler (Bio-Rad, United States). The mixture was separated on a 4% polyacrylamide gel in 0.5× Tris-Borate-EDTA buffer, and the gel images were taken with an Odyssey R Infrared Imaging System (LI-COR, United States).

### Dual-luciferase reporter assays

We used a dual LUC reporter assay system to analyze transcriptional activity in rice protoplasts. First, the promoter of *SNAC1* was inserted into the LUC reporter vector pGreen II 0800, which includes a Renilla LUC (REN) gene driven by CaMV35S as an internal control. Then the pRTVcIPA1-HA vector was used as an effector. The reporter and effector plasmids were co-transformed into the protoplasts through the method described above. The transformed protoplasts were incubated in the dark for 36 h at 28 ° C. LUC and REN activity was measured according to the instructions of the Dual Luciferase Reporter Assay Kit (Vazyme, China). The min35s promoter was used as a negative control. The binding of *IPA1* to the candidate gene promoters was expressed as the LUC/REN ratio. All experiments were repeated in three biological replicates.

### Measurement of physiological characteristics

The hydrogen peroxide (H_2_O_2_) was stained using DAB, malondialdehyde (MDA), catalase (CAT) and peroxidase (POD) activity were measured according to the manufacturer’s protocol (Beijing Solarbio Science Technology Co., Ltd., Beijing, China). Rice leaves were taken at 10 days of drought treatment to measure CAT and POD activity, and at 14 days of drought treatment to measure MDA content.

## Results

### Expression patterns of *IPA1*

**Fig. 1:**
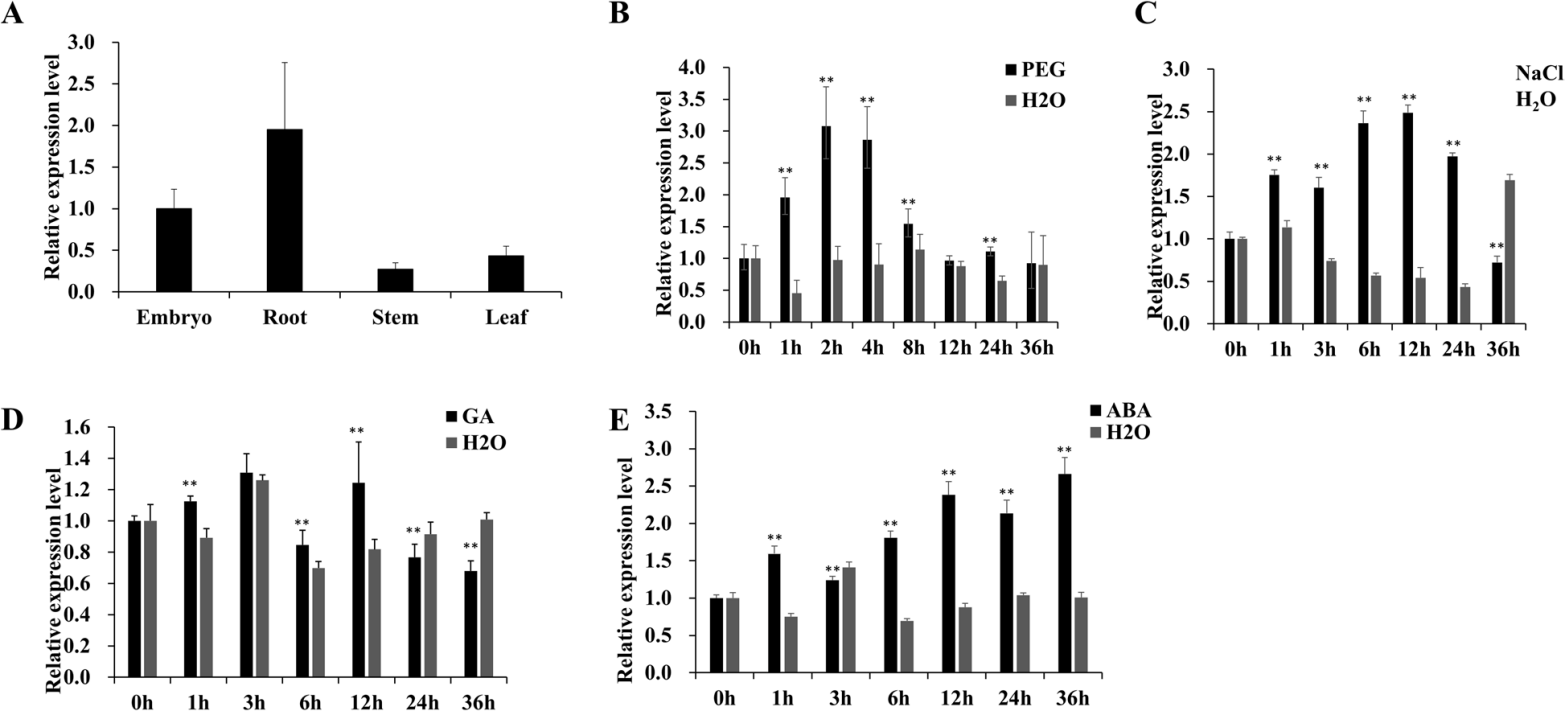
Expression patterns of *IPA1*. **A** Tissue-specific expression of *IPA1* in rice. **B** *IPA1* expression under simulated drought with 20% PEG treament. **C** *IPA1* expression under 100 mM NaCl solution. **D**, **E** Expression of *IPA1* under GA and ABA. Data are means ± se (*n* = 3). Statistical significance was determined by Student’s t-test. *, *P* < 0.05; **, *P* < 0.01

We analyzed the expression profiles of four representative tissues (root, stem, leaf and embryo from seedlings). RNA was extracted from different tissues, and RT-qPCR was performed to determine the expression pattern of *IPA1*. *IPA1* was expressed in seedlings in all tissues, with the highest levels in the roots (Fig. 1a).

We also assessed whether and how *IPA1* contributes to the responses to abiotic stress and hormone treatment. The *IPA1* transcript levels increased significantly after polyethylene glycol (PEG) 6000 and NaCl treatments (Fig. 1b,c), after hormone treatment, no significant improvement in IPA1 expression levels was observed after GA treatment, and the expression of *IPA1* peaked at 36 h after ABA treatment (Fig. 1d, e), thus indicating that the expression of *IPA1* varied in response to different abiotic stresses.

### Phenotype of *IPA1* knockout plants in the mature stage

**Fig. 2:**
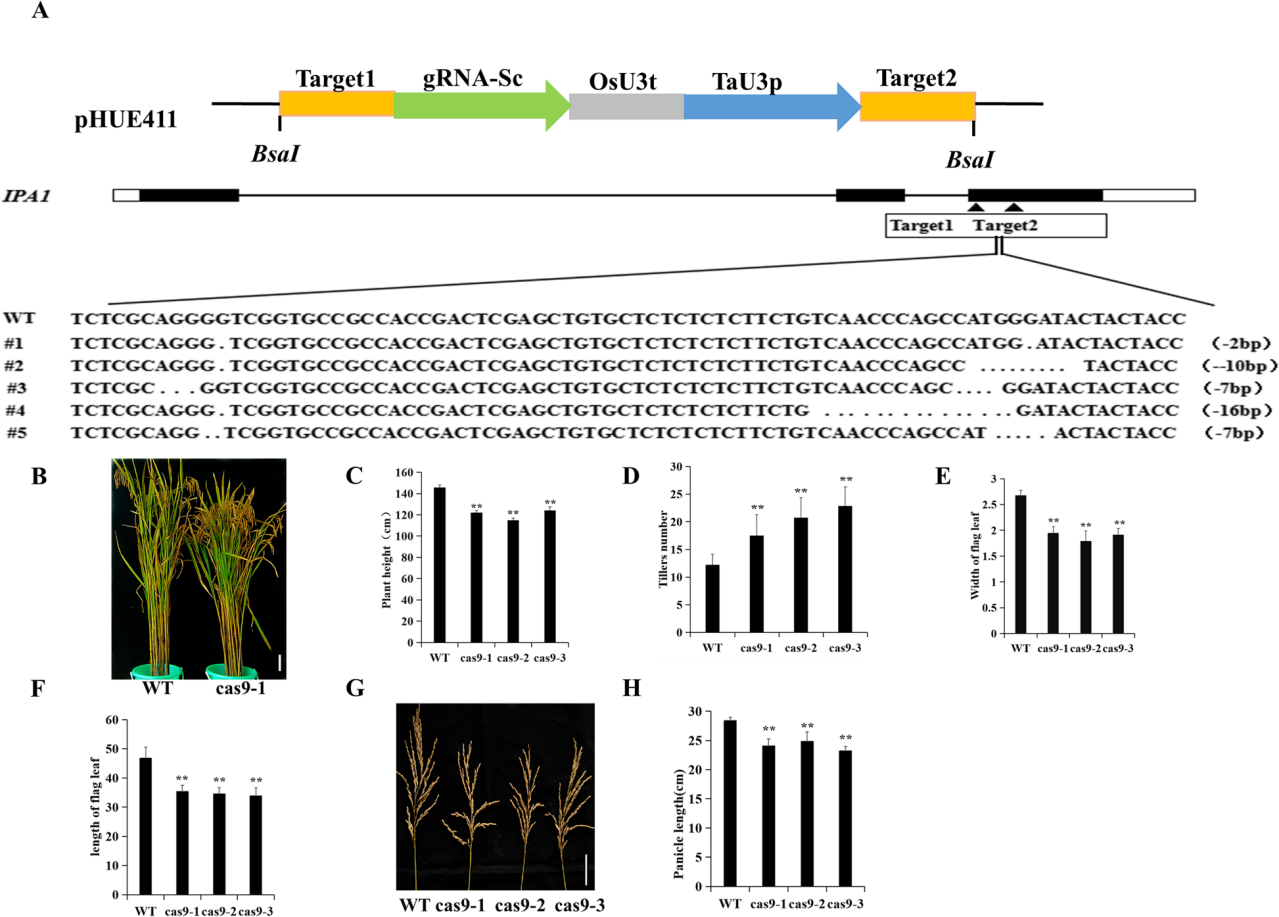
Phenotype of mature *IPA1* knockout plants. **A** Construction of CRISPR/Cas9 vector with pHUE411. Sequence analysis of mutation sites in *IPA1* knockout plants. Note:… are deleted bases; #1–#5 are knockout transgenic plants; and Target1 and Target2 are the targets for the design. Scale bar is 18 cm. **B** Phenotype of FH7185 and knockout transgenic plants. **C** Heights of FH7185 and knockout transgenic plants. **D** Numbers of tillers of FH7185 and knockout transgenic plants. **E** Flag leaf widths of FH 7185 and knockout transgenic plants. **F** Flag leaf lengths of FH7185 and knockout transgenic plants. **G** Ear lengths of FH7185 and knockout transgenic plants. Scale bar is 5 cm (**H**) Panicle lengths of FH7185 and knockout transgenic plants. Data are means ± se (*n* = 3). Statistical significance was determined by Student’s t-test. *, *P* < 0.05; **, *P* < 0.01

To investigate whether *IPA1* is associated with drought, we constructed a knockout vector with the pHUE411-CRISPR/Cas9 system and obtained positive transgenic plants, as mediated by *Agrobacterium* transformation (Fig. 2a), which were denoted Cas9–1, Cas9–2 and Cas9–3. Compared with the WT mature plants, the *IPA1* Cas9 plants showed dwarfism and more tillers (Fig. 2b, c, d), shorter and narrower flag leaves (Fig. 2e, f), and shorter panicles (Fig. 2g, h). The change in tiller number was opposite from that in plants with *IPA1* overexpression [28].

### Knockout of *IPA*1 is sensitive to drought stress

**Fig. 3:**
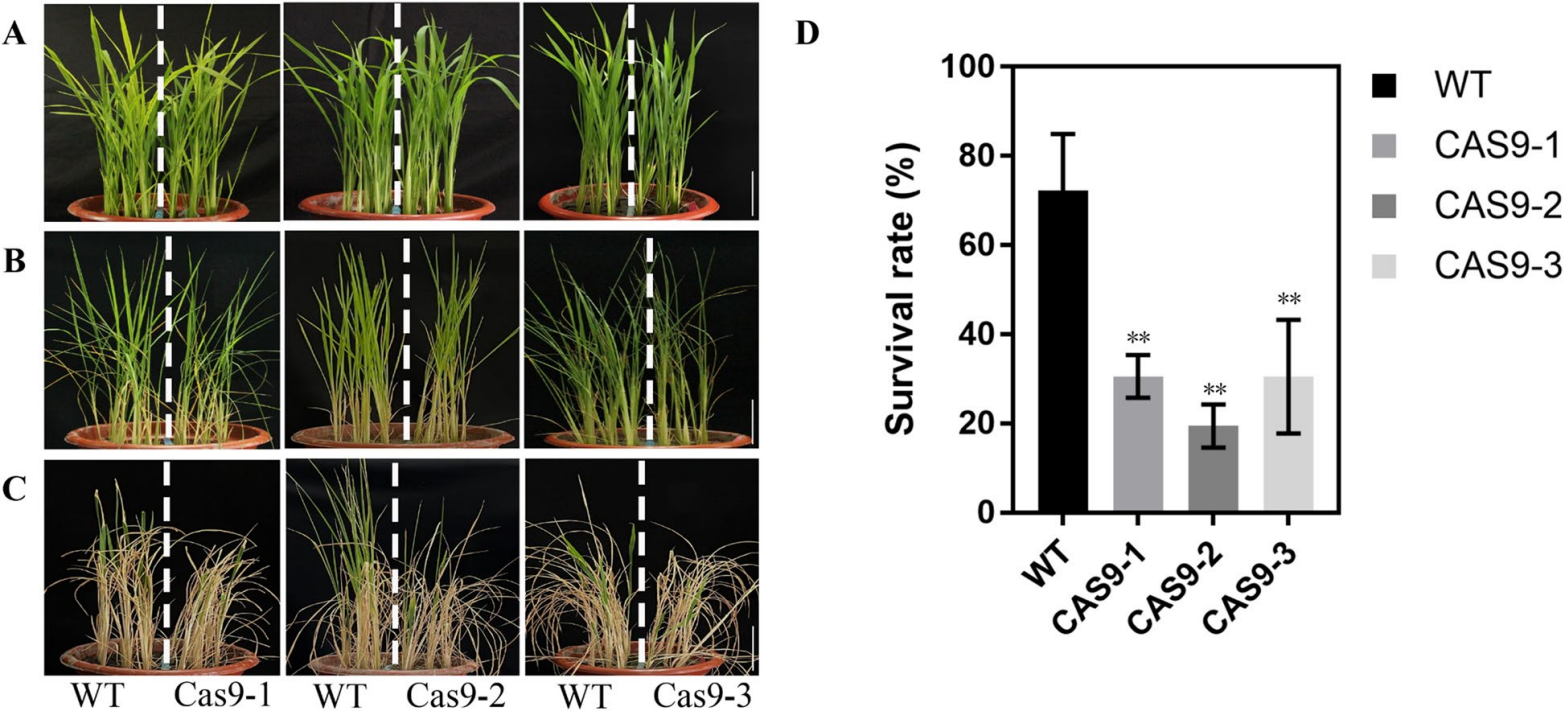
Drought tolerance of *IPA1* knockout seedlings. **A**, **B**, **C** Phenotypes of *IPA1* seedlings in dry soil conditions. Scale bar is 5 cm (**D**) Survival of *IPA1* under soil drought treatment. Data are means ± se (*n* = 3). Statistical significance was determined by Student’s t-test. *, *P* < 0.05; **, *P* < 0.01

The effects of drought stress on the performance of *IPA1* Cas9 plants were investigated and compared. Five-leaf stage plants were used for analysis. Under normal conditions, all lines grew normally, and no significant differences were observed (Fig. 3a). When water was withheld from these plants for 8 days, phenotypic changes were observed. Leaf rolling was substantially in *IPA1* Cas9 plants compared with WT plants (FH7185) (Fig. 3b). After 14 days of drought stress treatment, all knockout lines exhibited severe symptoms of drought stress and almost complete wilting, in contrast to their corresponding wild-type lines. After drought treatment, the plants were watered for recovery. Four days after watering resumed, the *IPA1* knockout became withered, as compared with the corresponding wild-type lines (Fig. 3c). After 14 days of watering, more than 72.2% of the WT (FH7185) lines recovered and grew normally. However, only 30.56, 19.44 and 30.56% of the *IPA1* knockout lines recovered (Fig. 3d), indicated that knockout of *IPA1* decreases drought tolerance at the seedling stage in rice.

### IPA1 directly activates the expression of SNAC1

NAC, basic leucine zipper (bZIP) and WRKY transcription factors are relatively well described families of transcription factors associated with resistance to abiotic stresses in plants [29–31]. To investigate how *IPA1* regulates drought tolerance, we selected transcription factors associated with drought resistance and other drought-associated genes from these three transcription factor families and analyzed the relative expression of these genes by RT-qPCR (Fig. S1a-e). The expression of NAC transcription factors was more variable and consistent with the trend in *IPA1* function-deficient transgenic plants and wild-type plants with significant differences. The expression of *OsWKRY1*3, *OsWRKY47*, *DMS2* and *OsbZIP71* significantly differed between the transgenic and wild type plants. We furtherly analyzed genes with high expression variation between *IPA1* overexpressing transgenic plants and MH86 plants. Only the *SNAC1*, *OsNAC10* and *OsNAC52* indicated the same trends and significant differences in expression between the transgenic plants with *IPA1* overexpression and MH86 plants (Fig. S1f, g). The yeast one hybrid results indicated that the *OsNAC10* and *OsNAC52* did not bind specifically to IPA1 (Fig. S1h, i).

**Fig. 4:**
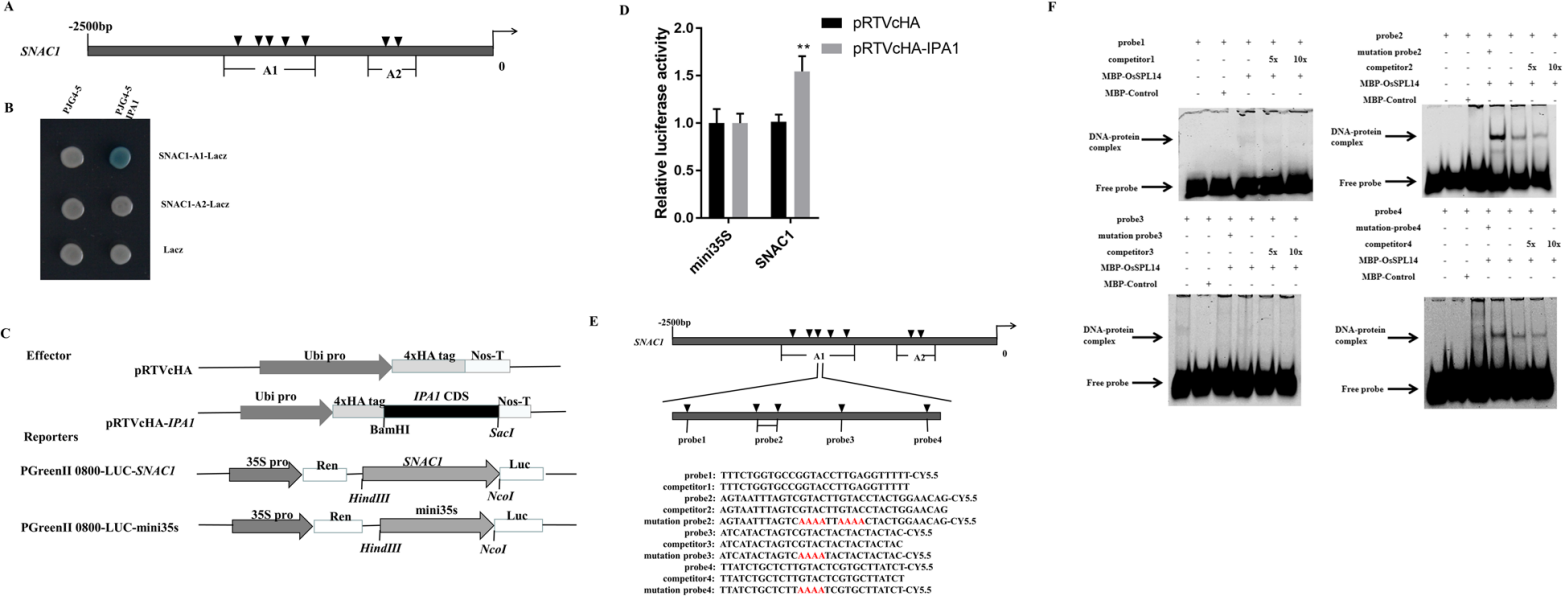
*IPA1* directly binds the promoter of *SNAC1*. **A** Segmented simplified map of the target genes; sequence is GTAC. **B** Interactions between *IPA1* and the promoter fragments of *SNAC1*, shown with a yeast one-hybrid assay. **C** The vector used in dual luciferase assays. **D** Detection of the luciferase activity of reporter genes. **E** Segmented simplified map of the target genes; sequence represents the GTAC motif. Sequences of segments: A1 and A2; probe sequence: probe-probe4; probe competitor: competitor; mutation probe: mutation probe; red bases: mutant bases. **F** Results of EMSA detection of IPA1 and SNAC1 promoters

SNAC1, a transcriptional factor belonging to the NAC family, regulates ROS homeostasis and confers drought tolerance in transgenic plants [32]. In addition, as a transcriptional activator, *IPA1* regulates its target genes by binding directly to the core motif GTAC or indirectly to the core motif TGGGCC/T of target gene promoters [16]. Bioinformatics analysis has identified seven GTAC motifs in the promoter of SNAC1 (Fig. 4a). We searched previously published ChIP-seq data for *IPA1* [16] and found that *SNAC1* is a potential target of *IPA1*, suggesting that the *IPA1* may directly activate the expression of *SNAC1*.

Therefore, we used yeast one-hybrid assays to investigate direct binding between IPA1 and the promoter of *SNAC1*. The *IPA1* directly binds the A1 promoter region of SNAC1 (Fig. 4b). Both fragments of the SNAC1 promoter contain GTAC motifs. In co-transfected into rice protoplasts, we used an IPA1-HA fusion protein driven by the ubiquitin promoter and a firefly LUC driven by the target gene promoter as the effector and reporter, respectively. The *IPA1* directly bound the GTAC motifs in promoter fragments of SNAC1. The transcriptional activity of LUC driven by the target gene promoter was significantly up-regulated in cells. The results suggested that *IPA1* interacts with the promoters of these two target genes rather than with the min35S promoter (Fig. 4c, d).

To further determine whether IPA1 specifically binds the GTAC motif, we performed EMSA. The double-stranded Cy5.5 labeled probes used in this experiment were synthesized according to the GTAC motif region, and unlabeled probes were used as competitors. IPA1 protein was successfully expressed in *E. coli* (Rosetta) after fusion with an N-terminal MBP tag. MBP-IPA1 bound GTAC motif labeled probes and formed DNA-protein complexes. Migration then decreased under increasing doses of competitive probes. The fusion protein could bind to the probe2 and probe4.Moreover, the specific binding of the fusion protein toprobe2 and probe4 was eliminated by addition of unlabeled competitors (Fig. 4e, f). Our results suggested that the *IPA1* directly binds GTAC in the *SNAC1* promoter region.

### IPA1 knockout rice plants are susceptible to drought stress

**Fig. 5:**
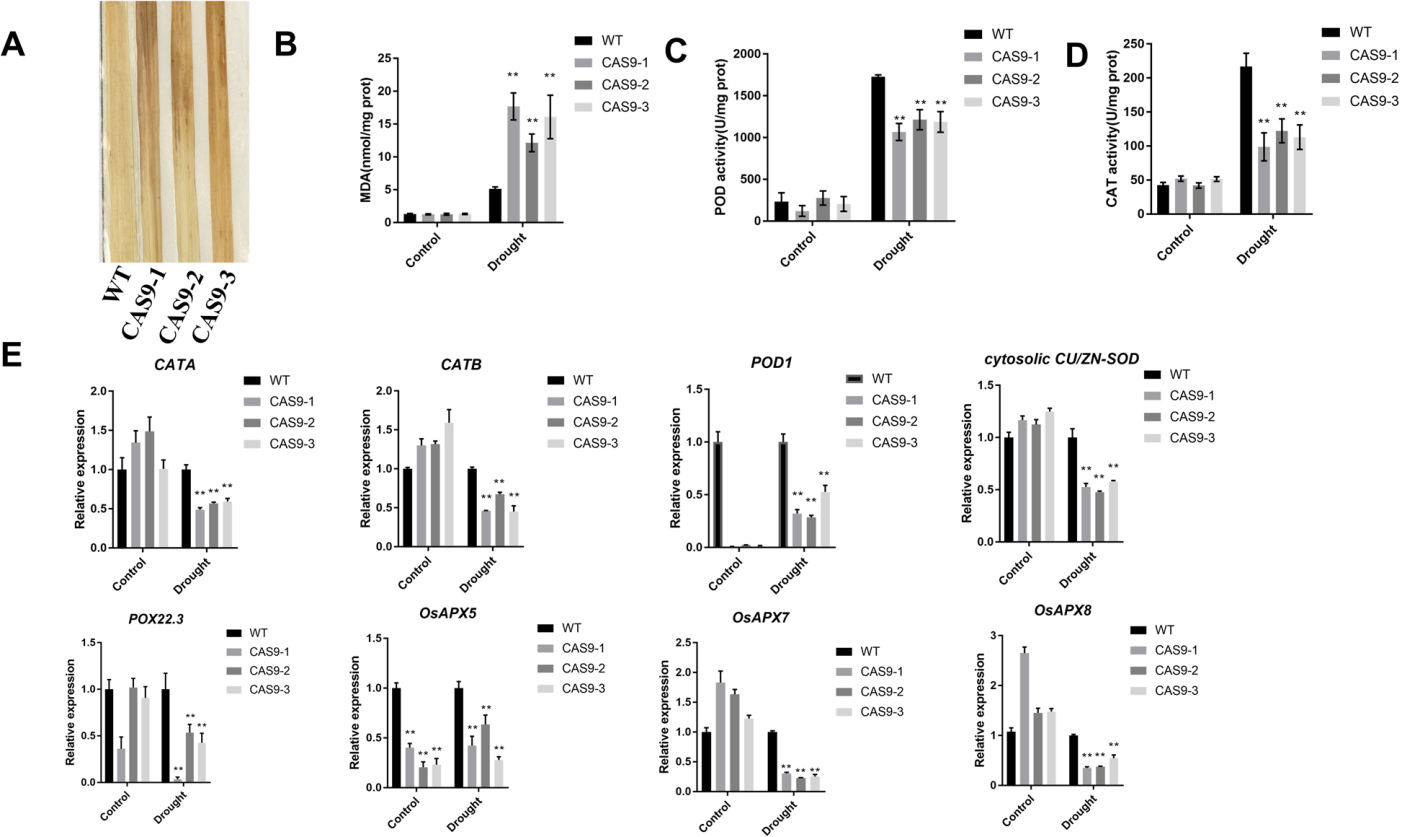
*IPA1* plants susceptible to drought stress in rice. **A** DAB staining for H_2_O_2_ in leaves from oxidative stressed WT and IPA1 mutant plants. **B**, **C**, **D** MDA, POD and CAT content of the WT and *IPA1* mutants during drought stress treatment. **E** Expression levels of ROS-related genes in WT (FH7185) and *IPA1*-CAS9 in response to drought stress. The relative expression levels of ROS-related genes were determined by qRT-PCR using gene-specific primer sets (Supplementary Table S1) and normalized to that of Actin150. Data are means ± se (*n* = 3). Statistical significance was determined by Student’s t-test. *, *P* < 0.05; **, *P* < 0.01

To investigate whether the *IPA1* promotes drought tolerance by regulating ROS homeostasis in rice, we treated WT and *IPA1*-Cas9 plants with drought stress and then used 3,3′-diaminobenzidine (DAB) staining to qualitatively detect H_2_O_2_ accumulation. The *IPA1*-Cas9 strain exhibited more H_2_O_2_ accumulation than the WT strain (Fig. 5a). Under normal growth conditions, MDA levels was similar between WT and all transgenic plants under drought stress, but was significantly higher *IPA1*-Cas9 than WT plants (Fig. 5b). These results suggested that knockout of *IPA1* significantly increased the excessive accumulation of ROS caused by drought stress.

The activity of CAT and POD was measured in 5-week-old seedlings under drought stress for 10 days. Under normal growth conditions, the POD and CAT activity did not appear to be significantly affected in *IPA1*-Cas9. Under drought stress, the POD and CAT activity of Cas9 plants significantly decreased (Fig. 5c, d). These results indicated that the role of *IPA1* in drought resistance may be associated with the antioxidant response to resist oxidative stress under drought. We further examined the transcript levels of a dozen genes associated with ROS scavenging. A total of eight genes, including two CAT-family genes (*CATA* and *CATB*), three APX-family genes (*OsAPX5, OsAPX7* and *OsAPX8*), a *POD1*, *POX22.3*and *Cu/Zn-SOD*, were expressed less in *IPA1* knockout plants than in WT plants under drought conditions (Fig. 5e). Enzymatic antioxidants, including catalase (CAT), and ascorbate peroxidase (APX), peroxidase (POD) and superoxide dismutase (SOD) are key ROS scavenging enzymes [33–35]. Altogether, these data provide a clear demonstration that knockout of *IPA1* might have reduced activity of ROS scavenging enzymes and thereby leads to lower expression of such important genes for reduced efficient ROS scavenging, may significantly contribute to excessive H_2_O_2_ accumulation and greater oxidative damage in the *IPA1* knockout plants, which is associated with the increased sensitivity of the plants to drought stress.

### Overexpression of *IPA1* increases the ROS-scavenging ability and decreases oxidative damage that are associated with increased drought tolerance

To elucidate the mechanisms of rice drought tolerance of *IPA1* in rice, the *IPA1*-OE vector was constructed and transformed into rice MH86. Three individual transgenic lines with single copy insertions, named as OE line 1, OE line 2 and OE line 3, were randomly selected for further study. qRT-PCR was carried out to detect the IPA1 transcript in transgenic plants. The expression level of IPA1 was significantly higher in the transgenic plants than in the WT (Fig. S2).

**Fig. 6:**
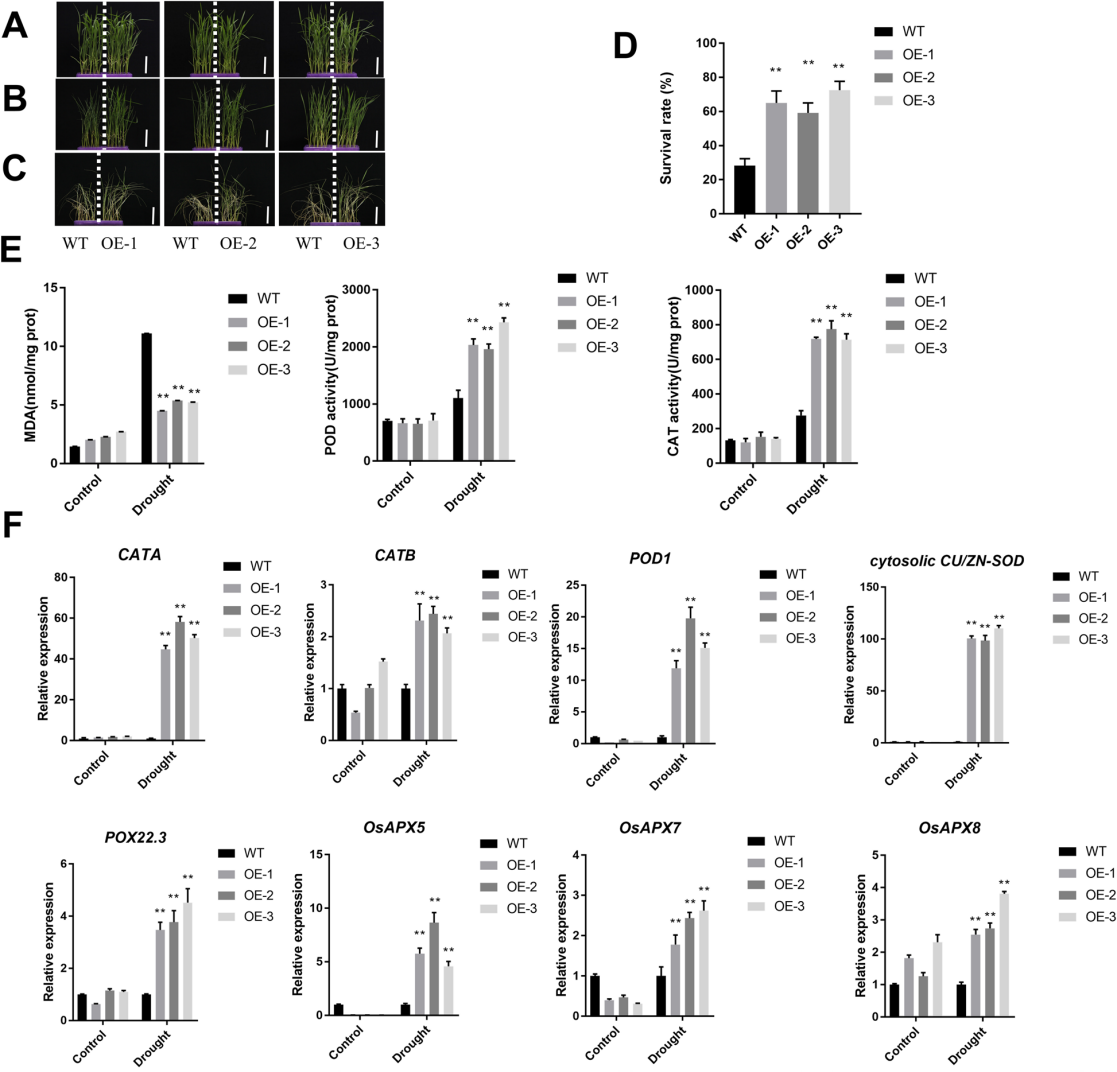
Overexpression of *IPA1* increases the ROS-scavenging ability and decreases oxidative damage that are associated with increased drought tolerance. **A**, **B**, **C** Phenotypes of *IPA1* seedlings in PEG treatment. Scale bar is 5 cm. **D** Survival of IPA1 under soil drought treatment. **E** MDA levels, POD and CAT activity of the WT (MH86) and *IPA1* overexpression during drought stress treatment. **F** Expression levels of ROS-related genes in WT (MH86) and *IPA1*-OE in response to drought stress. The relative expression levels of ROS-related genes were determined by qRT-PCR using gene-specific primer sets (Supplementary Table S1) and normalized to that of Actin150. Data are the means ± SD (*P < 0.05, **P < 0.01, Student’s t-test) of three technically independent experiments

Five-leaf stage plants were used for analysis. Twenty-five percentage of PEG treatments simulate drought stress. Under normal conditions, all lines grew normally, and no significant differences were observed (Fig. 6a). When water was withheld from these plants for 48 hours, the phenotypic changes were observed. The leaf rolling was substantially in WT plants compared with *IPA1* OE plants (MH86) (Fig. 6b). After 10 days of drought stress treatment, all WT lines exhibited severe symptoms of drought stress and almost complete wilting, in contrast to their corresponding the *IPA1*-OE lines. After drought treatment, the plants were recovery watered for four days, the wild-type lines became withered compared with the corresponding *IPA1*-OE lines (Fig. 6c). The results indicated that the ratio of the *IPA1*-OE plants survived was from 59.17 to 72.50%, while only 28.33% of the WT(MH86) plants survived under drought treatment (Fig. 6d).

To verify the important role of *IPA1* in drought resistance. We measured the MDA levels, POD and CAT activity of *IPA1*-OE plants and WT plants before and after drought treatment. Under normal growth conditions, the MDA levels, POD and CAT activity was not appeared to be significant difference compared to WT plants. Under drought stress, the MDA levels of *IPA1*-OE plants significantly decreased, and the POD and CAT activity of *IPA1*-OE plants significantly higher than those of WT plants (Fig. 6e). We furtherly examined the transcript levels of a dozen genes associated with ROS scavenging. A total of eight genes, including two CAT-family genes (*CATA* and *CATB*), three APX-family genes (*OsAPX5, OsAPX7* and *OsAPX8*), a *POD1* and *Cu/Zn-SOD*, were highly expressed in IPA1-OE plants than in WT plants under drought conditions (Fig. 6f). These results implied that the function of *IPA1* in drought tolerance may be associated with the enhanced antioxidant response to counteract oxidative stress under drought.

## Discussion

Drought stress is an important factor affecting crop production [1]. Drought stress causes leaf senescence, and affects root water uptake and various physiological processes [1, 36]. Previous studies have shown that the transcriptional factors not only regulate plant growth and development, but also play important roles in plant resistance to abiotic stresses [37, 38]. The SPL gene encodes a plant-specific transcription factor that plays a critical role in plant growth and development [4, 39]. Previous studies have shown that the *OsSPL* genes, such as *OsSPL3*, *OsSPL7*, *OsSPL14* and *OsSPL18*, increase tiller numbers [40]. IPA1/OsSPL14 is a transcriptional factor associated with the ideal plant architecture. *IPA1* expression confers an ideal plant architecture in rice, including fewer tillers, stronger culms (stalks), larger panicles and higher grain weight [15]. In this study, we used the CRISPR/Cas9 system to generate *IPA1* knockout mutants to characterize the role of *IPA1* in drought stress, and found that knockout of *ipa1* significantly decreased rice drought tolerance at the seedling stage. The mature *IPA1*-Cas9 plants demonstrated dwarfism and more tillers, shorter and narrower flag leaves, and shorter panicles compared with WT plants. Previous studies have shown that overexpression of *IPA1* decreases tiller number and yields a strong stalk [41, 42]. *IPA1* is most highly expressed in the roots of seedlings, and knocking down *IPA1* may affect root development and thus decrease the soil water uptake capacity and tolerance to drought stress.

Under drought stress, plants have evolved many stress-associated defense mechanisms, among which transcription factors are responsible for regulating the expression of a variety of stress-associated genes. The bZIPs are important transcriptional factors that regulate ABA signaling and drought response in plants [43]. *OsbZIP23* plays a key role in drought tolerance [44]. *OsbZIP16* and *OsbZIP71* positively regulate drought tolerance and the ABA signaling pathway [45, 46]. NAC transcriptional factors play important roles in regulating plant growth and the abiotic stress response [47]. Overexpression of *SNAC1* increases drought tolerance in rice [48]. *OsNAC2* regulates the drought stress response and ABA-mediated response [29]. Overexpression of *OsNAC52* is highly sensitive to ABA and increases drought tolerance in rice [49]. *WRKY* transcriptional factors play important roles in regulating the abiotic response in plants [31]. *OsWRKY47* mutants are more sensitive to drought than wild type [50]. Overexpression of *OsWRKY30* in rice significantly increases drought tolerance [51]. We previously selected many drought-tolerant genes for expression analysis in WT and *IPA1*-Cas9 plants (Fig. S1a-e). The eight genes, including *CATA, CATB, POD1, CU/ZN-SOD, POX22.3, OsAPX5, OsAPX7*, *OsAPX8* with significantly lower expression were used to expression analysis in those plants than the wild type. We analyze the expression of the above eight genes and found that only *SNAC1*, *OsNAC10* and *OsNAC52* showed significantly up-regulated expression with *IPA1* overexpressing and WT plants (Fig. S1f, g). Furtherly, we searched for previously Chip-seq data for *IPA1* published [16] and found that *SNAC1*, *OsNAC10* and *OsNAC52* were potential targets of *IPA1*. The yeast one hybrid results indicated that the *OsNAC10* and *OsNAC52* did not specifically bind *IPA1* (Fig. S1h, i).

*SNAC1* is a NAC transcription factor involved in the ROS regulatory pathway in rice [52]. Several stress-associated cis-acting elements are present in the NAC gene promoter [53]. Our results indicated that *IPA1* directly activated the promoter of *SNAC1* and significantly increased the promoter activity with the yeast one-hybrid and dual-luciferase assays (Fig. 4b, d). Further verification indicated that *IPA1* directly influenced the promoter activity of *SNAC1* by EMSA (Fig. 4f). *SNAC1* has been reported to be a key regulator in rice [54]. *SNAC1* regulates *OsPP18*, thus modulating ROS homeostasis through an ABA-independent pathway [52]. Meanwhile, our study demonstrated that *IPA1* regulates *SNAC1* and enhances drought resistance in rice, thus positively influencing drought and oxidative stress homeostasis by regulating ROS homeostasis.

Plant growth and development, and abiotic and biotic stress adaptation, are regulated by endogenous small signaling molecules. Among them, plant hormones such as ROS play important roles in the growth or death responses of many specific cells [55]. As a toxic byproduct of aerobic metabolism, ROS can damage plant cells through their high activity and toxicity. Drought stress causes excessive accumulation of ROS in plants [56]. ROS have a dual function in abiotic stress. In the presence of abiotic stress, intracellular ROS levels are low and when plants are exposed to abiotic stress, ROS levels increase activating stress pathways in the plant cell, when ROS act as a signaling sensor to activate defence mechanisms in the plant system. In severe abiotic high levels of ROS accumulation, the excessive production of ROS can be toxic to plant cells and eventually lead to plant death [57]. Some studies have shown that plants increase drought stress tolerance by regulating ROS homeostasis [24]. For instance, *OsEBP89* knockout increases the ability of rice to scavenge ROS and enhances tolerance to drought stress [58]. *OsRbohB* exerts drought resistance in rice by mediating ROS production [59]. In this study, *IPA1*-Cas9 seedlings exhibited more withering under soil drought stress (Fig. 2a-c). Significantly greater H_2_O_2_ and MDA accumulation was observed in the leaves of *IPA1*-Cas9 plants than WT plants (Fig. 5b). The diminished POD and CAT enzyme activity (Fig. 5c, d) in *IPA1*-Cas9 plants suggested that the decreased drought tolerance might have been due to excessive accumulation of ROS and higher levels of MDA, decreased accumulation of POD and CAT enzymes, and increased accumulation of H_2_O_2_. We obtained the opposite results in the *IPA1*-OE plants. Therefore, the function of *IPA1* in drought tolerance may be associated with the regulation of antioxidant capacity, thus suggesting that *IPA1* gene may act in response to drought stress by regulating the production of ROS.

To control ROS levels and protect cells under stressful conditions, plant tissues contain several enzymes to scavenge ROS (SOD, CAT, peroxidases and glutathione peroxidase and so on) [60]. However, excessive accumulation of ROS can lead to oxidative stress resulting in cellular damage or even death. Scavenging excess ROS can avoid or mitigate the damage to plant metabolism caused by stress and thus improve tolerance to drought [56]. Increased expression levels of genes encoding ROS detoxification enzymes successfully improve drought tolerance in plant [61]. In this study, after the drought treatment the expression levels of ROS detoxification-related genes *CATA, CATB, POD1, CU/ZN-SOD, POX22.3, OsAPX5, OsAPX7* and *OsAPX8* were significantly increased in *IPA1*-OE plants (Fig. 6f). In addition, the POD and CAT activities were significantly higher in *IPA1*-OE plants compared to WT plants (Fig. 6e).

In summary, our results indicated that the knockout of *IPA1* decreases drought resistance in rice seedlings and overexpression of *IPA1* increases drought tolerance in rice. The *IPA1* regulate the drought tolerance by affecting reactive oxygen species (ROS) content in rice. The Yeast one-hybrid, dual-luciferase and EMSA analyses indicated that *IPA1* directly activates *SNAC1* expression and directly affects tolerance to drought and oxidative stress through the regulation of ROS homeostasis. Our findings may have application value in increasing the drought resistance of rice.

### Statement

The study protocol complies with relevant institutional, national, and international guidelines and legislation. We have permission to collect “*Oryza sativa*” materials in this study.

## Supplementary Information

**Additional file 1: Fig. S1.** (A-G) Gene expression associated with the drought tolerance pathway. (H,I) Interactions between *IPA1* and the promoter fragments of OsNAC10 and OsNAC52, shown with yeast one-hybrid assays. Data are means ± se (*n* = 3). Statistical significance was determined by Student’s t-test. *, *P* < 0.05; **, *P* < 0.01. **Fig. S2.** Gene expression analysis of *IPA1* in WT and OE lines. Data are means ± se (*n* = 3). Statistical significance was determined by Student’s t-test. *, *P* < 0.05; **, *P* < 0.01. **Table S1.** Primers used in this study.

## Data Availability

The mutant and wild type plant used in the study are from Rice Research Institute, Fujian Academy of Agricultural Sciences, China are available from the corresponding author on reasonable request.
