# Supplementary material for: IPA1 improves drought tolerance by activating SNAC1 in rice
Source: BMC Plant Biol. 2023 Jan 25;23:55. doi: 10.1186/s12870-023-04062-9 (PMC9875436; doi:10.1186/s12870-023-04062-9)
Supplement: Supplementary file 1 — Additional file 1: Fig. S1. (A-G) Gene expression associated with the drought tolerance pathway. (H,I) Interactions between IPA1 and the promoter fragments of OsNAC10 and OsNAC52, shown with yeast one-hybrid assays. Data are means ± se (n = 3). Statistical significance was determined by Student’s t-test. *, P < 0.05; **, P < 0.01. Fig. S2. Gene expression analysis of IPA1 in WT and OE lines. Data are means ± se (n = 3). Statistical significance was determined by Student’s t-test. *, P < 0.05; **, P < 0.01. Table S1. Primers used in this study. [file 12870_2023_4062_MOESM1_ESM.docx]

Supplementary materials


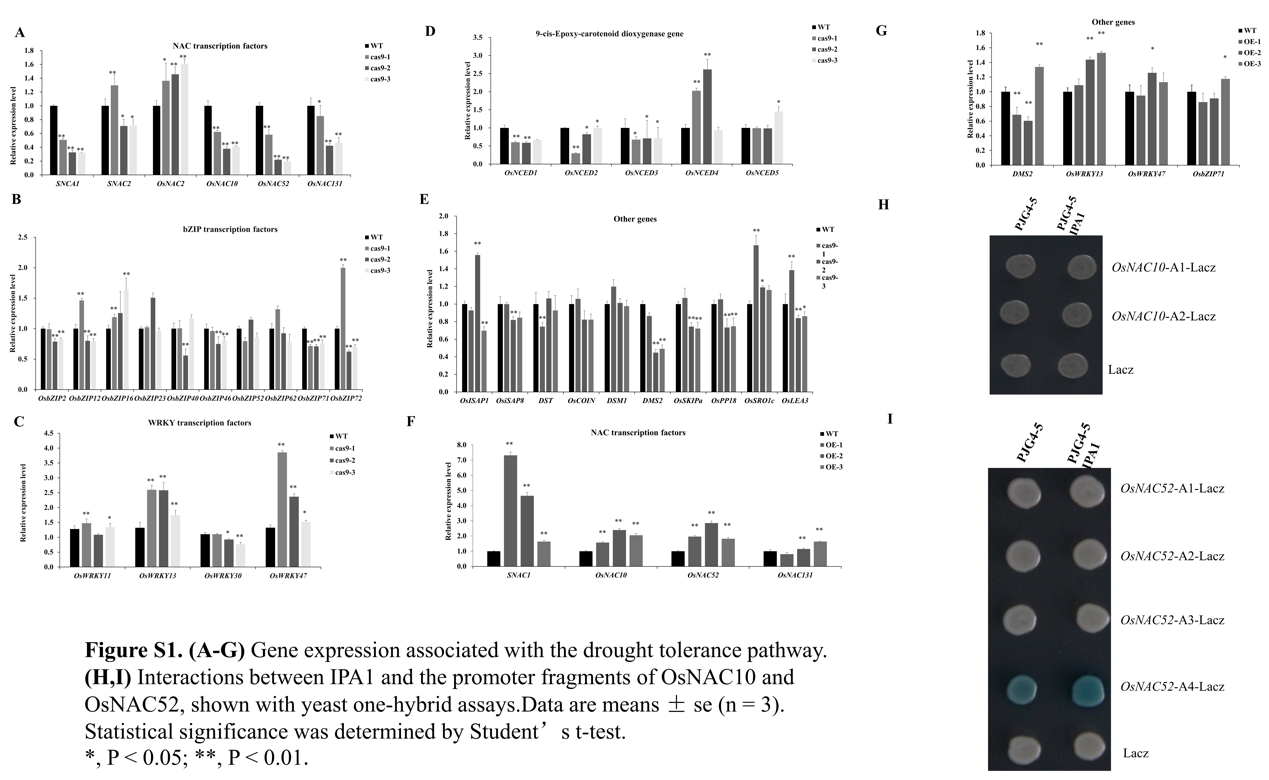


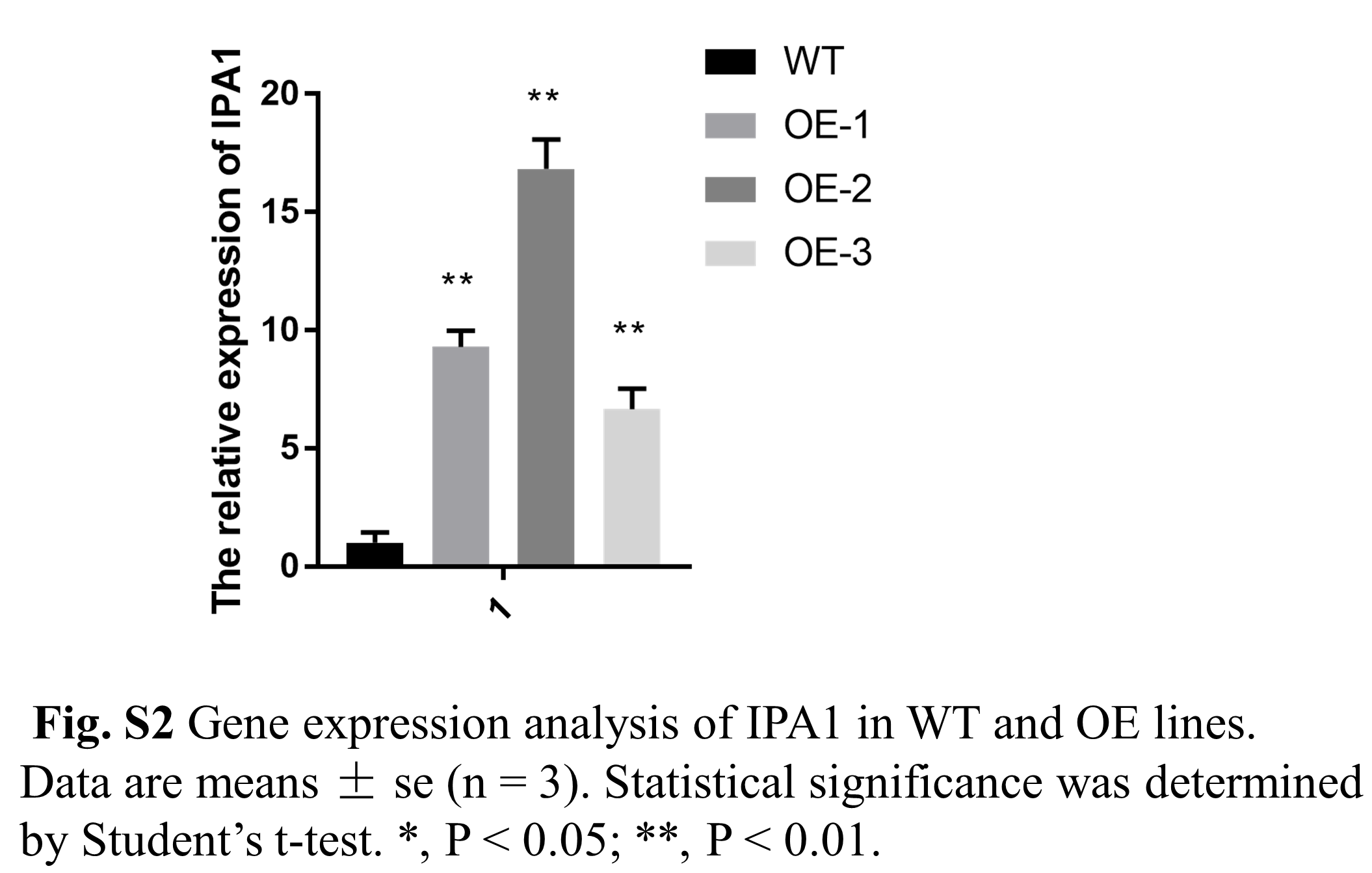


Table S1. Primers used in this study

| **Primer names** | **Sequences (5'to3')** |
| --- | --- |
| Cytosolic-CU/ZN-SOD-F | GGAAATGTCACCGCTGGAGAAG |
| Cytosolic-CU/ZN-SOD-R | AACGACGGCTCTGCCAATGATT |
| Actin150 F | TCGTCTGCGATAATGGAA |
| Actin150 R | CTCGTTGTAGAAGGTGTGATG |
| OsSPL14 F | AACCCCTTTGGCATCACGCTAC |
| OsSPL14 R | CCTTACGCTGCTTGGAACCCTT |
| OsPOD1-F | AACGCAACCACCAAGCCG |
| OsPOD1-R | CCTCGATCATGCCCATCTTGA |
| OsCAT1-F | GGACGAGGAGGTGGACTACT |
| OsCAT1-R | TGCTTGTGTATCGTCGCCTT |
| OsCAT2-F | ACGTCATGCTGAGAAGGTCC |
| OsCAT2-R | GACCGGTATCTCTCACCAGC |
| OsPOX22.3-F | CAGCTGCTCCAAGGTGAACTC |
| OsPOX22.3-R | AGATTTGCTTCCAGCAACGAA |
| OsAPX5-F | GGCGTCAGCTGCCGATGAACT |
| OsAPX5-R | ATGGCAAGCAGTAACACAGCTCA |
| OsAPX7-F | CAGGATCTCCTGGTCTTGCC |
| OsAPX7-R | TTGCACCCAGATTGCTCAGT |
| OsAPX8-F | CAACCAGAGCCCTTCGTCGCTG |
| OsAPX8-R | AGAGGCTTGTCCGGGCTGCC |
